# Social exclusion as a determinant of excess mortality in people with schizophrenia-spectrum and bipolar disorders: retrospective cohort study in 0.5 million people

**DOI:** 10.1017/S0033291725102110

**Published:** 2025-12-15

**Authors:** Jayati Das-Munshi, Lukasz Cybulski, Peter Byrne, Michael Dewey, Rosanna Hildersley, Sarah Markham, Craig Morgan, Robert Stewart, Milena Wuerth

**Affiliations:** 1 Department of Psychological Medicine, King’s College London, Institute of Psychiatry, Psychology and Neuroscience, London, UK; 2 Population Health Improvement UK (PHIUK), UK; 3 South London & Maudsley NHS Foundation Trust, London, UK; 4 ESRC Centre for Society and Mental Health KCL, London, UK; 5 Division of Insurance Medicine, Karolinska Institute, Stockholm, Sweden; 6 East London NHS Foundation Trust, London, UK; 7 Public Mental Health Implementation Centre, Royal College of Psychiatrists, London, UK; 8 Health Service and Population Research Department, Institute of Psychiatry, Psychology and Neuroscience, King’s College London, London, UK; 9 King’s College London, London, UK; 10 Centre for Anthropology and Mental Health Research in Action (CAMHRA), SOAS University of London, London, UK

**Keywords:** bipolar affective disorders, health inequalities, mortality, Schizophrenia, severe mental illness, social determinants, social exclusion

## Abstract

**Background:**

People with severe mental illness (SMI) (schizophrenia-spectrum and bipolar disorders) experience a 15–20-year reduction in life expectancy. The role of social determinants, including that of social exclusion, in contributing to excess mortality in SMI remains underexplored.

**Methods:**

Retrospective cohort study, comprising 8098 people with clinician-diagnosed SMI, matched to 581,209 population controls, followed for 5.7 years using person-level linked health/ census records. A social exclusion index was derived from census indicators: marital status, social isolation, economic inactivity, education, tenure, housing stability, and material assets.

**Results:**

Social exclusion was more common in SMI than in controls and strongly associated with higher mortality. Relative to the least socially excluded controls, adjusted hazard ratios (aHR) for mortality in SMI were: 16–44 years: aHR 7.58 (95% CI: 2.75–20.86) in the least socially excluded, increasing to 12.34 (7.92–19.24) in the most excluded; 45–64 years: 3.34 (1.98–5.64) [least excluded] increasing to 6.58 (5.32–8.14) [most excluded]; 65+ years: 2.71 (1.90–3.86) [least excluded], increasing to 3.07 (2.48–3.80)[most excluded]. Excess mortality among those with SMI was pronounced at younger ages if never married; by mid-life if living alone or economically inactive; and at 65+ years in those with SMI living alone, renting, or with no car ownership. Economic inactivity and lack of qualifications accounted for 16–35% of SMI mortality.

**Conclusions:**

Social exclusion is an under-recognized contributor to premature mortality in SMI. Targeting social determinants through novel socially-focused interventions could improve survival in people with SMI.

## Introduction

People diagnosed with schizophrenia-spectrum and bipolar affective disorders, known as severe mental illnesses (SMI), experience considerable reductions in life expectancy, ranging from 15 to 20 years (Wahlbeck et al., 2011). Physical health and health-related risk factors have been implicated (Firth et al., 2019; Saha et al., 2007), alongside a failure of integrated services and poorer quality care, linked to the lack of parity of esteem between mental and physical health (Firth et al., 2019; O’Connor et al., 2023). The inequity has widened over time (Saha et al., 2007).

People living with SMI may be more likely to be marginalized through processes operating via social exclusion. Social exclusion has been identified as a key determinant of health, with socially excluded groups experiencing marked health inequalities, including excess mortality (Aldridge et al., 2018). Although conceptually difficult to measure, social exclusion has been defined as an *“enforced lack of participation in key social, cultural and political activities”* (Morgan et al., 2007) which is linked to relational processes and although associated with material deprivation, is not purely characterized by poverty (Cuesta et al., 2024; Morgan et al., 2007; Popay, 2010). People with SMI are more likely to experience social exclusion, potentially perpetuated by stigma and discrimination (Morgan et al., 2007; Thornicroft et al., 2009). In the general population, social exclusion has been found to be associated with adverse mental health outcomes (van Bergen et al., 2018). Social isolation, a part of the underlying social exclusion construct (Cuesta et al., 2024; Morgan et al., 2007), is also associated with adverse physical health and mortality in the general population (Green et al., 2018; Holt-Lunstad et al., 2015). We could not identify any studies which have directly assessed the association of social exclusion with mortality in people with SMI, although there is some prior evidence suggesting that social isolation may be associated with excess mortality in mental disorders (see Supplementary Table 1 for reviewed evidence).

Our approaches to tackling excess mortality in people with SMI have been hampered by a lack of empirical evidence relating to social determinants and related to this, understanding the impact of social exclusion on mortality in SMI (O’Connor et al., 2023). In recent research on risk factors from the Global Burden of Disease (GBD) studies, the investigators also highlighted a gap in the evidence base relating to understanding social determinants as risk factors for health and mental health outcomes (GBD 2021 Risk Factors Collaborators, 2024; The Lancet Pyschiatry, 2024). Therefore, to address this gap in knowledge, we utilized a novel large-scale dataset, consisting of routinely collected clinical data from secondary mental health services, linked to the 2011 census of England and Wales, and linked to mortality records, which also contained a non-SMI general population control sample (Cybulski et al., 2024). We aimed to assess the association of SMI with all-cause mortality, and to quantify the association according to the presence of key social determinant indicators, including the presence of social exclusion.

## Methods

### Study design, linked datasets, participants

We undertook a retrospective cohort study which was assembled by curating linked data across three datasets. These included electronic health records (EHRs) linked to the census for England and Wales 2011 at person-level, linked to death certificate information, and linked to a census (non-SMI) control population.

EHR-derived information for the study was extracted from the South London & Maudsley (SLaM) NHS Foundation Trust, one of Europe’s largest mental health service providers, covering a catchment of 1.3 million people in London, UK. The Clinical Record Interactive Search (CRIS) was established in 2007 and is an ethically approved interface that enables researchers to access de-identified patient data for approved projects (Perera et al., 2016). Using CRIS, we ascertained individuals aged 15 to 90 diagnosed with schizophrenia-spectrum or bipolar affective disorders before the census (23 March 2011).

The census is a national mandatory data collection exercise which occurs across the United Kingdom every ten years. During the census, individuals in private households and communal establishments complete short self-report questionnaires detailing their social and demographic information. We matched individuals with SMI identified through CRIS, with population controls sampled from the neighboring catchment, identified through census (Cybulski et al., 2024). Population controls were ascertained as having no current or prior care from the mental health service provider by the time of census.

Population controls had to be resident in the same catchment area as the SMI sample (i.e. across the four southeast London boroughs of Lambeth, Southwark, Croydon, and Lewisham) and not have had any contact with the secondary mental health service provider. In the original study design, ~5 population controls were selected per clinical case under the care of SLAM, with mortality data also linked for SLAM cases and population controls. In this way, the linked sample represented a cohort study design, whereby clinical cases from SLAM Trust were matched to population controls from the same catchment area and then followed over time until death, or the end of the study. The final sample as described in this report, comprised a smaller number of cohort members with SMI matched to population controls, which is outlined in the study STROBE flow diagram (see Supplementary Materials, Figure 1).

All individuals in the cohort (comprising people with SMI and non-SMI population controls) were linked to the Office of National Statistics (ONS) mortality registration data (Cybulski et al., 2024).

### Measures

#### Psychiatric diagnoses

Clinician-ascribed diagnoses according to the International Classification of Mental Disorders 10th edition (ICD-10) (World Health Organization, 2011) were captured using structured fields supplemented by validated Natural Language Processing (NLP) algorithms across the free-text (Das-Munshi et al., 2021; Perera et al., 2016). ICD-10 diagnoses for F20-F29 (schizophrenia, schizotypal and delusional disorder) and F30-F31 (bipolar affective disorders) were used to identify individuals with SMI. We excluded individuals if they were diagnosed with dementia//organic brain disorder (ICD-10: F00-F09) prior to the SMI diagnosis.

### Measures

#### Sociodemographic indicators

Information on self-ascribed sex was obtained from 2011 census. Self-ascribed ethnicity was derived from the census and categorized according to the Office for National Statistics classification system, into: ‘Black Caribbean’, ‘Black African’, ‘Black Other’, ‘White and Black Caribbean’, ‘Irish’, ‘White British’, and ‘White Other’. Bangladeshi, Indian, or Pakistani ethnicity was combined into a ‘South Asian’ category, due to small numbers. The latter group included people identifying their ethnicity as ‘White and Asian’. Individuals who did not map to these groups were categorized as ‘Other ethnicity’.

#### Social exclusion measure

There is no consensus on how social exclusion should be measured (Cuesta et al., 2024; Morgan et al., 2007). Social exclusion occurs when individuals are prevented from fully participating in ‘economic, social, political and cultural life’ in society by external forces, rather than choice (Cuesta et al., 2024; Morgan et al., 2007). Previous assessments of social exclusion have included measures for material wealth as well as indicators specific to social participation (Cuesta et al., 2024; Morgan et al., 2007). Material indicators of socioeconomic deprivation on their own are not sufficient to encapsulate social exclusion (Cuesta et al., 2024). Social exclusion is a multi-dimensional, dynamic construct, capturing the relative impact of inequities on individuals (Cuesta et al., 2024).

Based on a conceptual framing (Morgan et al., 2007) (also see Supplementary Tables 1 and 8), we developed a list of indicators from the census 2011 that captured experiences of ‘social participation’ and ‘material wealth’ dimensions of social exclusion. For social participation which captured elements of ‘relational exclusion’ we used: marital status, from a question asking if respondents were currently single (never married or registered in a civil partnership), currently living alone (indicative of social isolation (Holt-Lunstad et al., 2015)), employment status/economic inactivity (social participation in the workforce (Huxley & Thornicroft, 2003; Sen, 2000) and also an indicator of material exclusion and/or labour marker exclusion) and highest level of qualifications (social participation in education (Dykxhoorn et al., 2024) and also an indicator of material exclusion). For material wealth indicators (Dykxhoorn et al., 2024) (also indicators for material exclusion), we used: a measure for housing wealth (home ownership vs. rental), housing stability (residing in a bedsit, hotel, caravan, shared house or flat vs house), and material wealth (car ownership). Employment status was obtained by cross-referencing participants’ reported occupation and economic activity in the census. People indicating they had worked for less than 15 hours that week were classified as “economically inactive.” We derived an education measure from a question asking about the highest level of qualifications achieved, which comprised “No educational qualifications” or “Any educational qualifications.” Each social exclusion measure was categorized into a binary measure.

### Mortality

A linkage to the Office for National Statistics (ONS) death certificate information was used to ascertain if cohort members had died during the study, with the date of death (Office for National Statistics, 2024).

#### Statistical analyses

We undertook survival analysis, with cohort members entering the study on the census date (March 23, 2011). Participants were followed until death, or end of the study on 13 December 2016, whichever came first. We produced descriptive statistics, deriving mortality rates per 100,000 person years. Cox proportional hazards regression was used to assess the hazard ratio for the association between baseline social exclusion indicators/index in 2011 and subsequent all-cause mortality. Age was handled as a time-varying covariate, using Lexis expansion to derive three age groups (15–45, 46–64, & 65+ years), leading to rates and hazard ratios stratified by age group. To determine if the risk of death associated with social exclusion varied according to SMI diagnosis, we fitted interactions between social exclusion indicators and the presence of SMI. Sex and ethnicity-adjusted hazard ratios for each combination of exposures (SMI status by social exclusion indicator) were generated, stratified by age. Age stratification was necessary to avoid violation of the underlying proportional hazards assumptions. We assessed the proportional hazards assumption by reviewing log–log plots, plotting Schoenfeld residuals, and testing for non-zero slopes.

We developed inverse probability weights to adjust for potential bias caused by higher levels of non-matching to the census, for people in contact with mental health services (Cybulski et al., 2024). Lower non-matching to census may be due to a number of factors, including a lower likelihood of census response and/or higher levels of residential instability, leading to missed matches by postcode (Cybulski et al., 2024). The inverse probability weights corrected for this potential selection bias and were incorporated across analyses, with the weight set to 1 for non-SMI controls.

We conceptualized social exclusion as the presence of multiple adverse social exclusionary experiences, which are experienced relatively less frequently in the population as a whole (Cuesta et al., 2024; Morgan et al., 2007; Sen, 2000). We used principal components analysis (PCA), assigning larger weights to indicators of social exclusion more unequally distributed in the population (Vyas & Kumaranayake, 2006). PCA using the correlation matrix, whereby one component was specified, was then used to derive a single unidimensional linear index of social exclusion. The final index was grouped into fifths.

### Sensitivity analyses

To assess the impact of missing data, we undertook multiple imputations with chained equations, under Missing at Random (MAR) assumptions. Ten imputation cycles were undertaken. The imputation regression contained the outcome (all-cause mortality), all regression covariates, interactions, and auxiliary variables (migration status, self-rated health, and self-rated disability). Estimates were derived by combining across imputed datasets, using Rubin’s rules.

We also calculated e-values to assess the potential role of unmeasured confounders (VanderWeele & Ding, 2017).

Our measure for ‘economic inactivity’ comprised those who reported working less than 15 hours a week and included students and retirees. Whilst these two groups are ‘economically inactive’, students participate in education, and retirees may have previously held occupations indicative of workforce participation. Therefore, we re-ran analyses excluding students and retirees from the ‘economically inactive’ group as a separate sensitivity analysis.

Statistical analyses were undertaken in STATA 18. The protocol was pre-registered on Open Science Framework (protocol: https://archive.org/details/osf-registrations-2q9rk-v1).

#### Involvement of people with lived experience

The study design and analyses were discussed with a service user and carer advisory group (SUCAG), established to inform upon research arising from the data linkages used in this study. One of the coauthors of this manuscript (SM) was a member of the SUCAG and was subsequently more closely involved in the development of this manuscript for publication. The input of the SUCAG and SM informed conceptual framing, selection of variables, and interpretation of the results.

## Results

The cohort consisted of 8,098 individuals with an SMI diagnosis and 581,209 individuals in the population control group, without a recorded contact with the Mental Health Trust (Supplementary Figure 1). All indicators of social exclusion were more common in the SMI group, compared to the control group (Table 1). Supplementary Tables 2–3 display mortality rates per 100,000 person years, associated with social exclusion indicators, stratified by age.

**Table 1.** Demographic characteristics of the cohort

	SMI diagnosis	
	Yes	No
	*N* = 8098 (%)	*N* = 581,209 (%)
Sociodemographic variables		
Age at census, years (mean, SD)	45.6 (13.9)	42.1 (15.5)
Sex		
Male	4101 (50.6)	285281 (49.1)
Female	3997 (49.4)	295928 (50.9)
Ethnicity		
White British	4033 (49.8)	271046 (46.6)
White Other/Irish	746 (9.2)	89084 (15.3)
Black Caribbean	1525 (18.8)	65266 (11.2)
Black African	873 (10.8)	66268 (11.4)
South Asian	465 (5.7)	38696 (6.7)
Chinese/Arab/Other/Mixed ethnicity	456 (5.6)	50849 (8.7)
Social exclusion indicators		
Marital/relationship status		
Currently/previously married or civil partnership	3313 (40.9)	316478 (54.5)
Never married/civil partnership	4785 (59.1)	264731 (45.5)
Social isolation		
Does not live alone	5401 (66.7)	508105 (87.4)
Lives alone	2697 (33.3)	73104 (12.6)
Economic inactivity		
Employed	2211 (27.3)	399932 (68.8)
Economically inactive	5887 (72.7)	181277 (31.2)
Highest qualification		
Any educational qualification	5983 (73.9)	498522 (85.8)
No educational qualifications	2115 (26.1)	82687 (14.2)
Housing status (owner-occupier)		
Owns in some capacity/lives rent free	2612 (32.3)	295363 (50.8)
Rents	5486 (67.7)	285846 (49.2)
Housing stability		
House	3075 (38.0)	288334 (49.6)
Bedsit/hotel/caravan/house share/flat	5023 (62.0)	292875 (50.4)
Car ownership		
1+ cars owned	2856 (35.3)	359732 (61.9)
No cars owned	5242 (64.7)	221477 (38.1)

**Figure 1.**
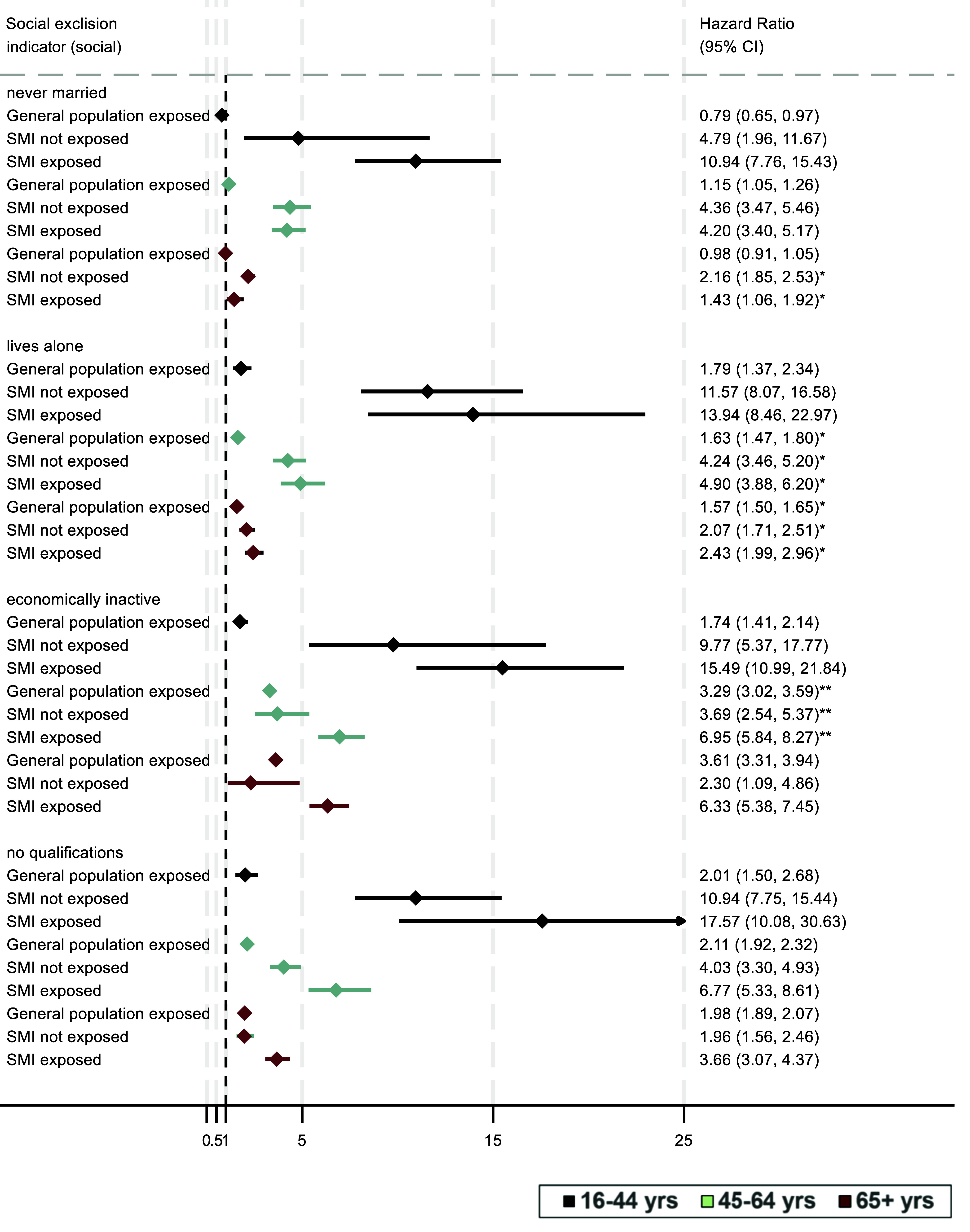
Association of social exclusion (social participation indicators) with all-cause mortality. **Key:** Displayed estimates are weighted, stratified by age (black: 16–44 yrs, green: 45–64 years; maroon: 65+ years) and adjusted for sex and ethnicity; Reference group across all models is the general population not exposed to any of the displayed social indicators for that age group, shown as the dashed reference line of 1.00. *p* values for interaction:***p < 0.001; **p < 0.01; *p < 0.05.

**Figure 2.**
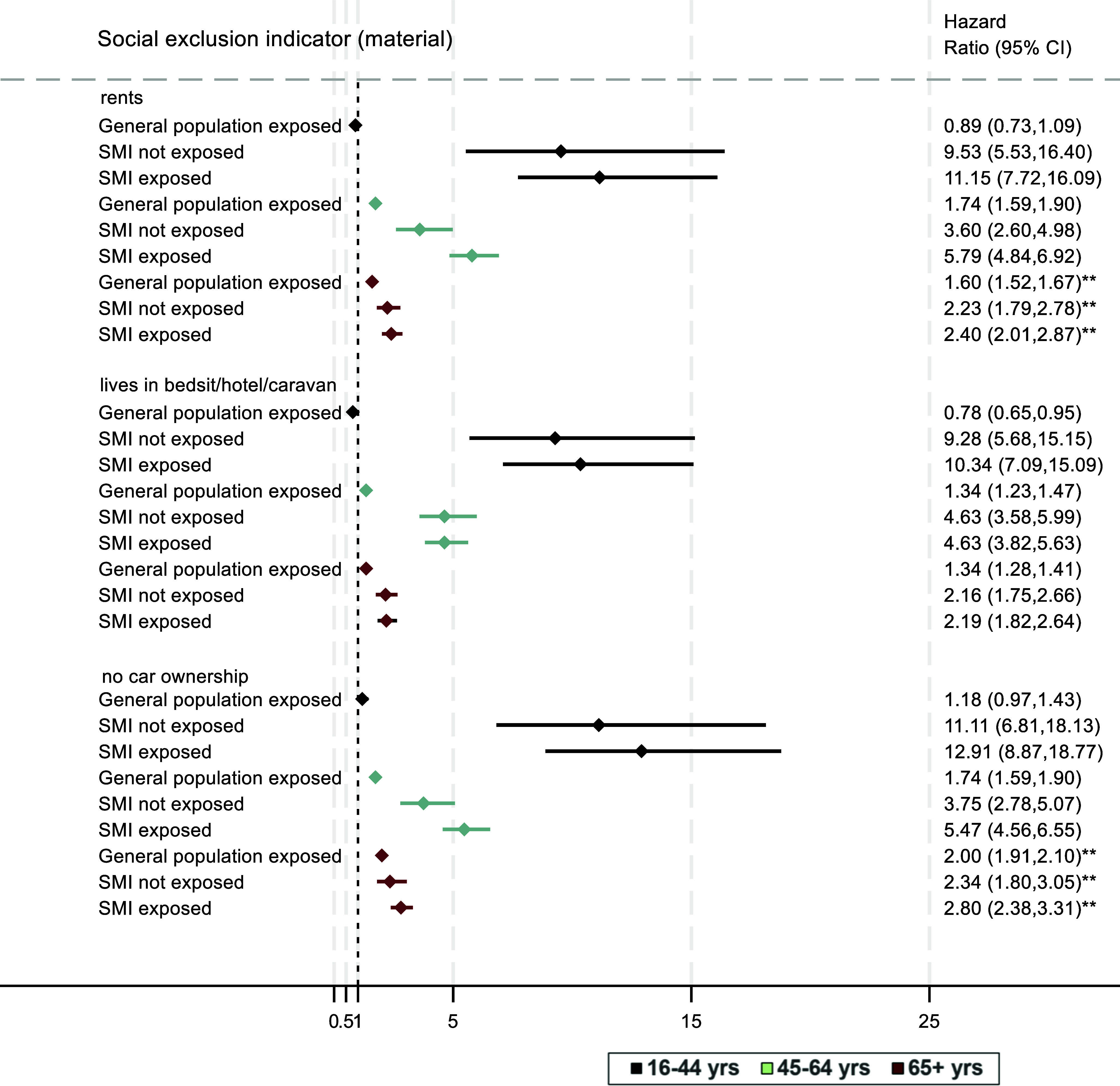
Association of social exclusion (material wealth indicators) with all-cause mortality. **Key:** Displayed estimates are weighted, stratified by age (black: 16–44 yrs, green: 45–64 years; maroon: 65+ years) and adjusted for sex and ethnicity. Reference group across all models is the general population not exposed to any of the displayed social indicators for that age group, shown as the dashed reference line of 1.00; *p* values for interaction:***p < 0.001; **p < 0.01; *p < 0.05.

Figures 1 and 2 display the associations between social exclusion indicators and all-cause mortality. Models were stratified by age, adjusted by ethnicity and sex, and displayed relative to the general population control group not exposed to the social exclusion indicator. All estimates were weighted to account for records not matching in the original linked dataset. In the younger groups, irrespective of social exclusion, rates of mortality were high in those with SMI compared with the general population, with a low absolute number of deaths. For each indicator of social exclusion, the highest mortality risk was in the SMI group exposed to the indicator, particularly in the youngest groups. For the two older age groups, the pattern of higher mortality in SMI for people experiencing social exclusion was, in general, similarly evident. There was strong evidence in support of statistical interactions across multiplicative models for marital status (in the youngest and oldest age groups), living alone (middle and oldest age groups), economic inactivity (the middle age group), rental tenure (oldest age group), and lack of car ownership (oldest age group).

**Figure 3.**
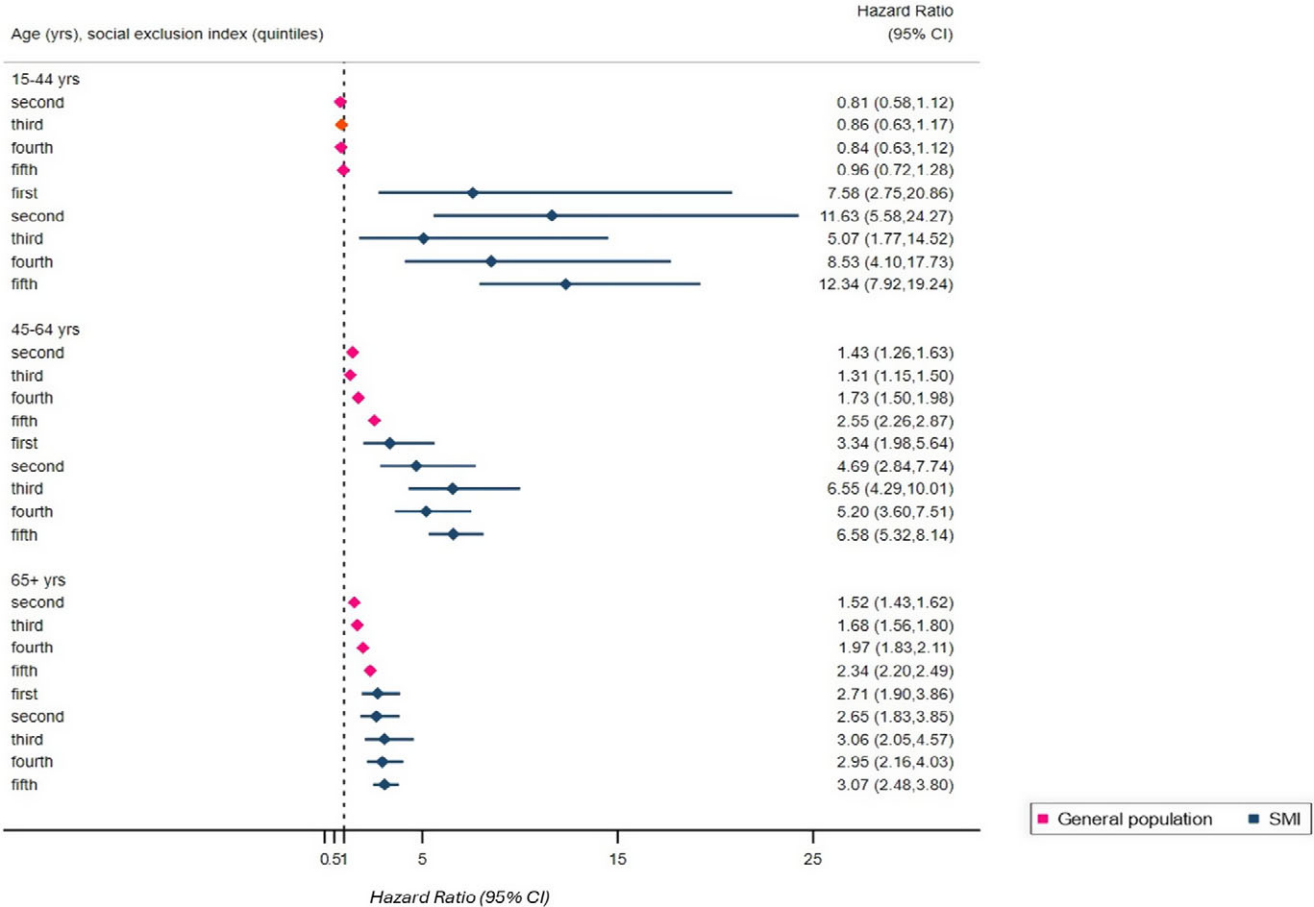
Association of social exclusion index with mortality. Social exclusion index quintiles range from 1 (lowest social exclusion) to 5 (highest social exclusion). Displayed Hazard Ratios are relative to the reference (general population in the lowest/first social exclusion quintile). Displayed estimates are weighted and have adjusted for sex and ethnicity.

Figure 3 displays the association of the social exclusion index with all-cause mortality. The reference group for these models was the general population in the least socially excluded fifth of the index. Whereas there was no association of increasing levels of social exclusion and death in the general population compared to the reference at age 15–44 years, an increased risk of death in people with SMI was already evident in this age group. An association between increasing social exclusion and death was evident at all other age groups, with a larger magnitude of association evident in the SMI group.

**Figure 4.**
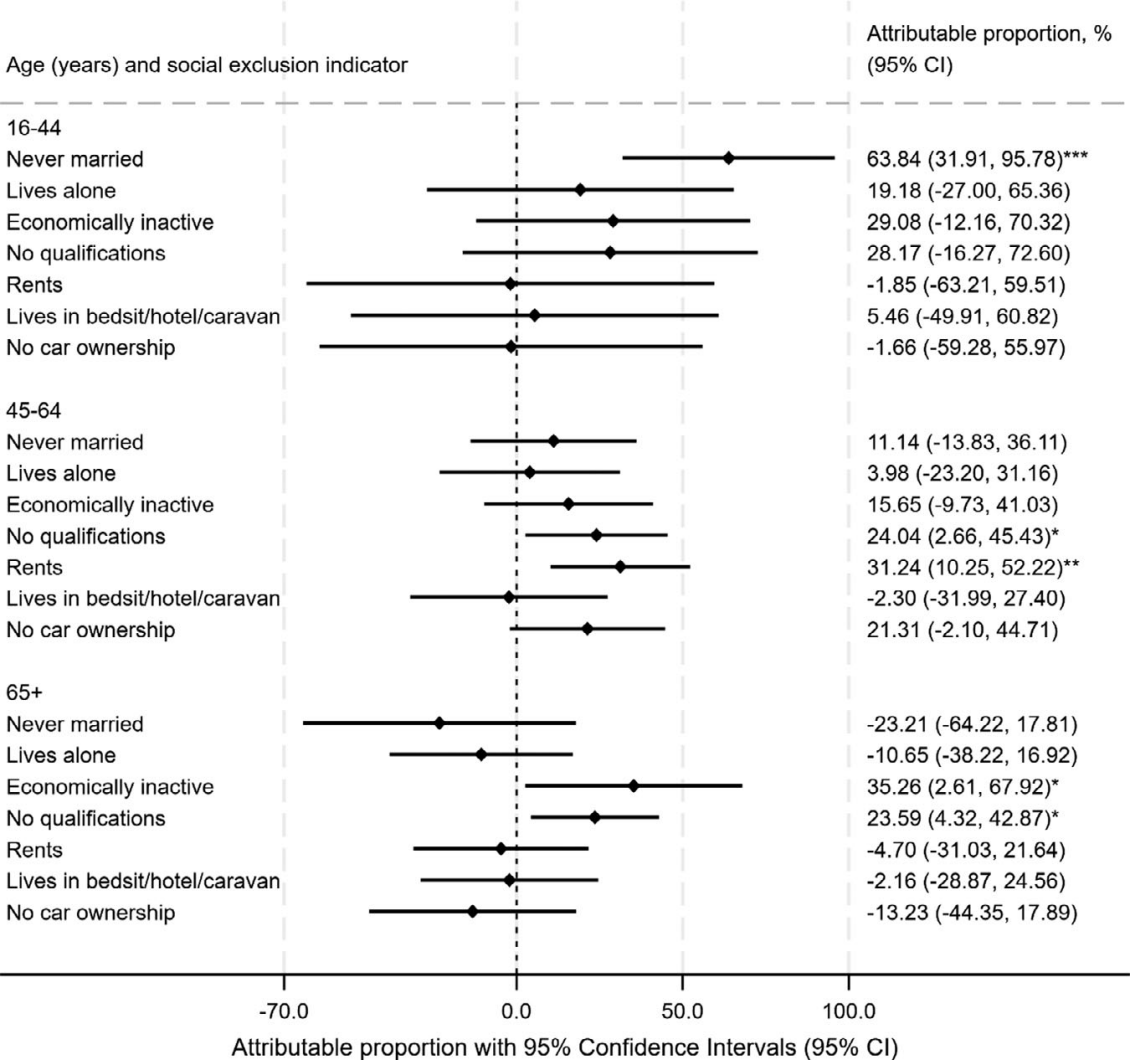
Attributable proportion (with 95% confidence intervals) for social exclusion indicators and all-cause mortality risk in severe mental illness. **Key:** *p* values for interaction:***p < 0.001; **p < 0.01; *p < 0.05; Displayed estimates are adjusted for sex and ethnicity and stratified by age.

Figure 4 presents attributable proportions and Supplementary Table 4 presents the relative excess risk of interaction (RERI), for the two-way interaction of individual social exclusion indicators with the presence of SMI, in additive models for the relative risk of all-cause mortality. In these analyses, an RERI of 0 indicates a ‘perfectly additive’ model, a negative RERI indicates a sub-additive model and a positive RERI indicates a super-additive model (Rothman et al., 2008). Super-additive associations were substantial for some of the social exclusion indicators, suggesting that a large proportion of the excess risk of all-cause mortality was attributable to the indicator in question. This was evident in the 15–44 year groups for never being married/civil partnership (attributable proportion 64%); at mid-life (45–64 years) for no qualifications (attributable proportion: 24%) or renting (vs owner-occupier) (attributable proportion 31%), and in older adults (65+ years) for no qualifications (attributable proportion 24%), and in those with economic inactivity (attributable proportion 35%). Across all ages, there was evidence of super-additive associations for no qualifications (28%, 24%, 24% at ages 16–44, 45–64, 65+ respectively) and economic inactivity (29%, 16%, 35% at ages 16–44, 45–64, 65+ respectively), although for some of the estimates 95% CIs were wide, necessitating a degree of uncertainty around interpretation. Supplementary Table 5 displays unadjusted and age/sex adjusted associations.

### Sensitivity analyses

Unweighted estimates for all associations (Supplementary Figures 2–3) suggested a slightly smaller association with social exclusion indicators and all-cause mortality. Sensitivity analyses with multiple imputation did not reveal large differences between imputed estimates and unweighted estimates (Supplementary Figures 4–5). E-values for the associations between SMI (exposed and not exposed to social exclusion indicators) and mortality were large, suggesting that results were robust to potential unmeasured confounding (Supplementary Table 6) (VanderWeele & Ding, 2017). Sensitivity analyses removing students and retirees from the ‘economically inactive’ variable did not have a large impact on associations. Specifically, removal of these two groups from the ‘economically inactive’ variable led to a slightly larger effect size in the youngest group (age 16–44 years) and slightly smaller effect size in the oldest groups (age 65+ years), and almost no change for the group at mid-life (45–64 years) (see Supplementary Table 7).

## Discussion

In this large population cohort, we found high levels of social exclusion in people living with SMI. Social exclusion was associated with excess mortality in this population. In addition, we found that each aspect of social exclusion, spanning material exclusion to social exclusion from relationships, was also associated with excess mortality in SMI. Economic inactivity and a lack of education were consistently associated with elevated mortality in people with SMI, at all ages. In addition, we found that never being married/or being in a civil partnership in the youngest age group was associated with high attributable proportions for death, potentially indicating the importance of close relationships at this age. It is possible that the relationship dimension of social exclusion plays an important role in minimizing suicide risk as well as deaths from natural causes. This will be explored in future work.

On the social exclusion index, we found that even in the youngest age group with SMI, social exclusion was linked to higher mortality—an association not observed in the general population. As social exclusion increased, so too did mortality risk, particularly in midlife and in older adults with SMI. These findings highlight the urgent need to address this health determinant early in this population.

There has been a dearth of research on social exclusion and mortality. Most prior studies assessing mortality in people with SMI have relied on healthcare records, where it is challenging to assess social exclusion, as these indicators are usually missing or poorly collected. In the present study, we circumvented this through utilizing an innovative data linkage between electronic health records, the census, and death records (Cybulski et al., 2024). One previous study identified an association between social disconnection and mortality in men but not women with mental disorders (Laustsen et al., 2024). Our study builds on this work by assessing how other elements of social exclusion, such as unemployment, educational exclusion, and material disadvantage, are associated with mortality. Our findings also shed light on this important issue, specifically for people with schizophrenia-spectrum and bipolar disorders, in whom overall proportions of social exclusion were also markedly elevated, and in whom we found associations with mortality from relatively younger ages (15 years and onwards).

Despite a well-established association between social determinants and health outcomes, the lack of empirical evidence on how social determinants are associated with excess mortality in severe mental illnesses (SMI) has been striking (O’Connor et al., 2023). Our study addresses this critical gap in knowledge and has relevance to the *Global Burden of Disease* (GBD) initiative (GBD 2021 Diseases and Injuries Collaborators, 2024). The GBD initiative quantifies the impact of health conditions globally, taking into account disability and premature mortality. Recent GBD analyses highlighted the importance of 88 risk factors for health outcomes (GBD 2021 Risk Factors Collaborators, 2024). Although this was a comprehensive analysis of risk factors for health, assessments of social risks were notably scarce in the report (GBD 2021 Risk Factors Collaborators, 2024). The authors of this report noted that mental health conditions were “responsible for 5.4% of the global burden, but only 8% were attributable to risk factors” (GBD 2021 Risk Factors Collaborators, 2024). Commentators have therefore highlighted an urgent need to understand how socioeconomic determinants are risk factors for mental illness and death (The Lancet Pyschiatry, 2024), which our current analyses have now started to address for severe mental health conditions. Outside of social exclusion, we also found that the component indicators of the social exclusion construct, which included marital status, social isolation, economic inactivity, low education, and lack of material asset ownership (car/home ownership), were associated with excess mortality in this population. In future GBD assessments, including measures similar to those used in the present study, could help to inform this critical gap in knowledge.

The findings provide a compelling rationale for future complex intervention development for physical health to incorporate approaches to improve employment and education outcomes in people with SMI, particularly as there is already a good evidence base for employment interventions in people with SMI (Bond et al., 2023; Modini et al., 2016). Employment has multiple benefits beyond economic gains, including a sense of purpose, increased social networks, less physical inactivity, more agency, increased social status, better healthcare access, and combating stigma. Potential solutions addressing the harms of stigma-exclusion-inequities, will need to be multi-level, coproduced, and across multiple domains. On an individual level, this could entail integrated care approaches, with a focus on considering social context as part of routine clinical care (Greenburgh et al., 2025). Peer support interventions, to enhance social inclusion/community integration and reduce social isolation/disconnection, could also be explored (Greenburgh et al., 2025). The complex challenges of premature mortality in SMI will need complex solutions, which acknowledge how inequities and exclusion interact, for this neglected population.

Social exclusionary processes have been described as multidimensional and relational, operating through stigma (anticipated/experienced) (Thornicroft et al., 2009), as well as processes linked to power and equity (Popay, 2010) and are not just a result of being materially disadvantaged or impoverished (Cuesta et al., 2024). Specifically, exclusionary forces may operate across a range of interlinked domains and across multiple levels (individual/household/area level) (Cuesta et al., 2024; Dykxhoorn et al., 2024; Popay, 2010). Exclusionary processes lead to an *“unjust distribution of resources, capabilities and rights*” which are then associated with observed health inequalities (Popay, 2010). In keeping with this literature, our findings confirm that for people with SMI, social exclusion may be experienced across multiple domains, at all ages. As has been previously highlighted, “*social exclusion does not just happen, it is a sequela of socially patterned structural forces*” (O’Connor et al., 2023). Social exclusion leads to people with SMI having limited opportunities to develop supportive relationships, gain education and competitive employment, which may in turn lead to relative material disadvantage (Cuesta et al., 2024). Our findings then suggest, ultimately, an association between experiencing social exclusion and subsequent mortality in people with SMI. The interplay of these factors with physical health, self-harm, and subsequent mortality may be through a range of mechanisms, including reduced help-seeking from anticipated stigma (Thornicroft et al., 2009), diagnostic overshadowing and ensuing reduced access to evidence-based treatments (Firth et al., 2019; O’Connor et al., 2023; Thornicroft et al., 2009), the direct adverse impacts of material disadvantage on health, health-related behaviors (diet, tobacco use, and physical activity) (O’Connor et al., 2023), and a heightened risk of suicide, through social isolation and a lack of supportive relationships (O’Connor et al., 2023). Stigmatizing processes, driven by a power differential, have additional multiple negative effects, including internalized stigma and a belief that some things (success, health checks, relationships) are for other, more ‘deserving’ people – the ‘why try’ effect of stigma (Corrigan et al., 2016).

Social disability (impaired relationships, an ability to live independently and work/school functional impairment) (Green et al., 2018) may arise from the cognitive impairments and/or negative symptoms due to schizophrenia. Conversely, in previous work, which specified the social defeat hypothesis (Selten et al., 2017), it had been suggested that experiences of social exclusion may precede the onset of psychosis and are mediated through mesolimbic pathways (Selten et al., 2017). In our study, we modeled the joint association of SMI with these indicators, without assuming temporality (cause/effect). Our findings indicate that both the presence of SMI diagnosis and the presence of social disability/social exclusion indicators were associated with excess mortality. Future work could explore the nature of a temporal association further.

Study strengths included the use of self-report measures for social exclusion, the large sample, and the length of longitudinal follow-up of the cohort. The sample was representative of a large urban area, which generalizes well to other metropolitan regions. Further advantages included the use of health records enabling ICD-10 clinician diagnoses, enhanced through Natural Language Processing (NLP) algorithms, which have been validated (Das-Munshi et al., 2021). The use of population controls from the neighboring area would have ensured that shared neighborhood characteristics that could have confounded estimates were controlled. Sensitivity analyses indicated that findings were unlikely to have been accounted for through unmeasured confounding. In previous work, we have observed that people with SMI are less likely to have a matched census record, which might be because they are less likely to take part in the census (Cybulski et al., 2024). To address this, we used inverse probability weights to correct for this potential bias.

There were some limitations. There are varying methods for measuring social exclusion (Morgan et al., 2007), and the construct itself is contested (Morgan et al., 2007). It has been previously highlighted that social exclusion is a broad concept which is indexed by a range of domains, inclusive of ‘material exclusion’ alongside social relationships, and other types of social participation (see Supplementary Table 8). Indicators of poverty and relative material disadvantage may constitute ‘social exclusion’, but on their own are not enough to assume social exclusion (Cuesta et al., 2024; Morgan et al., 2007). Our approach was therefore informed by a framework which incorporated social participation and material indicators of exclusion; however, our measure did not include political participation and digital exclusion (Dykxhoorn et al., 2024). Conceptually, the variables which we included in our assessments captured salient elements of this underlying construct for people living with SMI (as also outlined in Supplementary Table 8) (Morgan et al., 2007), although other elements of social exclusion could be explored in the future.

We did not assess physical health and health-related behaviors (smoking, alcohol use, diet, and exercise), although it is likely that these mediate the association between social exclusion and mortality (Green et al., 2018). Our methodology to identify people with SMI was based on case-ascertainment through clinician diagnoses in health records, so it is possible that the population control group may have included people with SMI if undiagnosed individuals had not made contact with the mental health service provider. However, the likely impact of this is that we would have under-estimated associations. The conceptual framing of our work was an assumption that social exclusion is a result of being unable to participate in society. Although our approaches to measuring this construct were in keeping with methodologies that have previously been used (Cuesta et al., 2024; Dykxhoorn et al., 2024; Morgan et al., 2007), a limitation of our study was that we could not directly assess the element of ‘choice’ around participation. Given prior literature that there may be gender specific associations (Laustsen et al., 2024), in future work, we plan to extend these analyses to examine interactions with gender and associations with specific causes of death (e.g. suicide vs deaths from natural causes). Future analyses could explore whether the association between the relationship dimension of exclusion and suicide risk is also seen at other ages and among women, where these deaths may not predominate. We also plan to explore associations with ethnicity in future work.

In conclusion, our findings provide compelling evidence that social exclusion, a construct which captures material disadvantage alongside barriers to participation in broader society (such as in relationships, education, and the workforce), could be a major, underrecognized contributor to premature mortality in SMI. Many of the social exclusion indicators identified in this study are modifiable (Greenburgh et al., 2025), for example, Individual Placement Support (IPS) to support improved employment outcomes in people with SMI (Bond et al., 2023). Our findings suggest that these approaches could also be trialed for improving physical health and mortality outcomes in this group. Our results underscore the possibility of novel targets for action, which could draw upon public health and integrated social-psychiatric interventions, to ultimately tackle the inequitable gap of early deaths in people with SMI.

## Supporting information

Das-Munshi et al. supplementary material Das-Munshi et al. supplementary material

## Data Availability

For mental health records (South London and Maudsley Trust deidentified data)- Data are owned by a 3rd party SLaM BRC CRIS tool, which provides access to anonymized data derived from SLaM electronic medical records. CRIS data can only be accessed by permitted individuals from within a secure firewall (i.e. remote access is not possible and the data cannot be sent elsewhere) in the same manner as the authors. The linked data in this study were covered by specific ethical agreements and access arrangements that restrict data sharing. Data supporting this study cannot be made available due to ethical issues. DOI for Open Science Framework (OSF) protocol: https://doi.org/10.17605/OSF.IO/2Q9RK.

## Supplementary material

The supplementary material for this article can be found at http://doi.org/10.1017/S0033291725102110.
