# Supplementary material for: Social exclusion as a determinant of excess mortality in people with schizophrenia-spectrum and bipolar disorders: retrospective cohort study in 0.5 million people
Source: Psychol Med. 2025 Dec 15;55:e375. doi: 10.1017/S0033291725102110 (PMC13058655; doi:10.1017/S0033291725102110)
Supplement: Das-Munshi et al. supplementary material [file S0033291725102110sup001.docx]

**SUPPLEMENTARY MATERIAL**

**Title:** Social exclusion, social disconnection, and excess mortality in people with schizophrenia-spectrum and bipolar disorders: Retrospective cohort study in 0.5 million people

**Authors:** Jayati Das-Munshi PhD, Lukasz Cybulski PhD, Peter Byrne MRCPsych, Michael E. Dewey PhD, Rosanna Hildersley MSc, Sarah Markham PhD, Craig Morgan PhD, Robert J. Stewart PhD, Milena Wuerth MSc

**Table of Contents**

[Figure 1. Flow of study participants 3](#_Toc209188562)

[Supplementary Table 1: Prior evidence on social exclusion and mortality in severe mental illnesses 4](#_Toc209188563)

[Supplementary Table 2: Crude death rate per 100,000 person years stratified by age band among individuals without an SMI 5](#_Toc209188564)

[Supplementary Table 3: Crude death rate per 100,000 person years stratified by age band among individuals with an SMI 6](#_Toc209188565)

[Supplementary Figure 2: Association of social exclusion (social participation indicators) with all-cause mortality; age, sex and ethnicity adjusted estimates, unweighted 7](#_Toc209188566)

[Supplementary Figure 3: Association of social exclusion (material wealth indicators) with all-cause mortality; age, sex and ethnicity adjusted estimates, unweighted 8](#_Toc209188567)

[Supplementary Figure 4: Association of social exclusion (social participation indicators) with all-cause mortality; age, sex and ethnicity adjusted estimates, multiple imputation 9](#_Toc209188568)

[Supplementary Figure 5: Association of social exclusion (material wealth indicators) with all-cause mortality; age, sex and ethnicity adjusted estimates, multiple imputation 10](#_Toc209188569)

[Supplementary Table 4: Relative excess risk due to interaction (RERI) for social exclusion indicators with severe mental illness, RERI estimates with 95% Confidence Intervals (95% CI) 11](#_Toc209188570)

[Supplementary Table 5: Association of severe mental illness (SMI) and social exclusion indicators with mortality, unadjusted (model 1) and age, sex adjusted associations (model 2) 12](#_Toc209188571)

[Supplementary Table 6: Sensitivity analyses, E-values* 13](#_Toc209188572)

[Supplementary Table 7: Sensitivity analyses, association between economic inactivity and mortality 17](#_Toc209188573)

[Supplementary Table 8: Developing a social exclusion indicator from census measures 18](#_Toc209188574)

## **Figure 1.** Flow of study participants

16,615 individuals in CRIS with relevant ICD-10 diagnoses: F20-F29 (schizophrenia, schizotypal and delusional disorder) F30-F31 (bipolar affective disorders)

52,376 individuals in CRIS with match to census record

108,629 individuals in secondary mental health care (CRIS), identified with a range of clinical psychiatric diagnoses

Exclusions:

- 8404 excluded because they were diagnosed after the study entry date on 23 March 2011.
- 596,124 population controls sampled from catchment of secondary mental healthcare provider, without a history of contact with mental health services and with census data, appended to the dataset.

604,528 individuals in appended dataset.

Exclusions:

- 248 individuals excluded because they died prior to the study start date.
- 4993 individuals excluded because they were below the age of 15 at the study start date.
- 2063 individuals removed because they were 90 or older the study start date.

597,224 individuals remaining in appended dataset.

Missing data:

- 7758 individuals (1.3%) excluded in general population sample due to missing data
- 159 individuals (0.03%) excluded in SMI sample due to missing data

581,209 individuals without a severe mental illness.

8,098 individuals with a severe mental illness.

## **Supplementary Table 1:** Prior evidence on social exclusion and mortality in severe mental illnesses

| **Evidence before this study**: To identify evidence to inform this research we undertook systematic searches for literature using the following databases and search terms: PubMed was searched for peer-reviewed articles published before November 29, 2024, with the search restricted to English language. The terms ‘mortality’, ‘death’, ‘decease*’, ‘died’, ‘dead’, or ‘remain alive*’ were paired with ‘social isolation’, ‘social exclusion’ ‘loneliness’, ‘living alone’, ‘material deprivation’, ‘deprivation’, ‘poverty’. Searches were restricted by terms: ‘schizophren*’, ‘psychosis’, ‘severe mental ill*’, ‘severe mental dis*’, ‘psychotic dis*’, ‘psychotic ill*’, ‘bipolar dis*’, or ‘manic depress*’.  Social exclusion was defined as occurring when individuals are prevented from fully participating in society by external forces, not by choice. Conceptually, social exclusion spans multiple dimensions which include participation in work, education, leisure and citizenship activities, and also includes social relationships, social isolation, as well as housing deprivation, poverty and material wealth. Whereas ‘inclusion’ denotes the involvement of people with severe mental illnesses in mainstream society, ‘social exclusion’ leads to people being excluded from mainstream society through underlying processes, such as stigma and discrimination.  We did not identify any studies which assessed the role of social exclusion with mortality in severe mental illnesses. However, three studies were identified which examined ‘social isolation’, which have been included under the broader social exclusion construct. In a previous study using data from the Danish Psychiatric Central Research Register, investigators assessed loneliness, low social support and social isolation, and found that between 24-61% of excess mortality in mental disorders could be explained by these indicators in men, but not in women. An older study examining 133 patients with schizophrenia from the US, did not find an association between social isolation and death. Finally, a study undertaken by our group identified that for racially minoritised people with SMI, living in areas of higher own group density, deaths from all-causes, natural causes and unnatural causes were lower. This was thought to be due to ethnically diverse areas buffering racially minoritised individuals against loneliness, social isolation as well as racialised exclusion, although these indicators were not directly assessed in this study.  **Added value of this study:** Although it is known that people living with severe mental illnesses experience high levels of social exclusion, the relationship of these experiences with mortality is unknown in this population. The unique dataset used in this analysis links person-level Census data with hospital records and death certificate information, which for the first time provides information on the self-reported indicators of social exclusion as experienced by a large cohort of individuals with severe mental illnesses compared to population controls. Our findings provide empirical evidence which confirms an association between social exclusion and mortality in people with severe mental illness. The findings suggest a need for active efforts to counter social exclusion, as experienced by people with SMI.  A lack of close relationships (never married or civil partnerships) in the youngest age group with SMI had a substantially elevated association with mortality, with marital status having an attributable proportion of 64% for mortality in this age group. At mid-life (45-64 years) and at older ages (65+ years) social isolation (living alone) in people with SMI was associated with a high excess risk of death. Economic inactivity and having no qualifications were also associated with high excess risk of death in people with SMI in these age groups. We developed an index of social exclusion which comprised all social exclusion indicators available in the linked dataset and took an approach which gave greater weight to those social exclusion indicators distributed more unequally in the population. We found that as social exclusion levels increased, so too did the risk of death. This was not evident in the younger age group (age 15-44 years) in the general population, although already evident in people with SMI. Our findings confirm, for the first time, the large and unequal effects of social exclusion on the risk of death in people with SMI, which starts early in life. Our findings have major implications in informing public health strategies to tackle this gap in life expectancy and suggest an urgent need to ‘move out of the clinic’ and consider broader social determinants, which incorporate social psychiatry and public mental health perspectives when developing interventions to address this inequity. |
| --- |

| **Supplementary Table 2:** Crude death rate per 100,000 person years stratified by age band among individuals without an SMI | | | | | | | | | | | |
| --- | --- | --- | --- | --- | --- | --- | --- | --- | --- | --- | --- |
|  |  |  |  |  |  |  | **Age band** |  |  |  |  |
|  |  |  |  | **15-44** |  |  | **45-64** |  |  | **65+** |  |
|  |  | ***Deaths (n, %)*** | | *428* |  |  | *2127* |  |  | *7811* |  |
|  | **N (%)** |  |  | **Rate (95% CI)** | |  | **Rate (95% CI)** | |  | **Rate (95% CI)** | |
| **Marital/ relationship status** |  |  |  |  |  |  |  |  |  |  |  |
| Currently or previously married | 316478 (54.5) | *8549* | *2.7%* | 28.5 (24.3-33.3) | |  | 243.3 (231.2-256.0) | |  | 2432.3 (2375.6-2490.3) | |
| Never married | 264731 (45.5) | *1817* | *0.7%* | 24.3 (21.5-27.3) | |  | 293.6 (271.9-317.1) | |  | 2521.1 (2361.7-2691.3) | |
| **Social isolation** |  |  |  |  |  |  |  |  |  |  |  |
| Does not live alone | 508105 (87.4) | *7142* | *1.4%* | 23.8 (21.5-26.4) | |  | 231.0 (220.1-242.5) | |  | 2130.4 (2073.0-2189.5) | |
| Live alone | 73104 (12.6) | *3224* | *4.4%* | 45.8 (35.9-58.5) | |  | 408.4 (373.8-446.3) | |  | 3399.5 (3273.0-3530.9) | |
| **Employment status** |  |  |  |  |  |  |  |  |  |  |  |
| Employed | 399932 (68.8) | *1938* | *0.5%* | 21.8 (19.4-24.6) | |  | 174.0 (164.1-184.5) | |  | 839.8 (772.9-912.3) | |
| Economically inactive | 181277 (31.2) | *8428* | *4.6%* | 36.6 (31.4-42.8) | |  | 531.9 (500.3-565.7) | |  | 2863.4 (2798.2-2930.1) | |
| **Qualifications** |  |  |  |  |  |  |  |  |  |  |  |
| Any education | 498522 (85.8) | *4974* | *1.0%* | 24.1 (21.7-26.6) | |  | 216.3 (205.6-227.5) | |  | 1705.6 (1646.5-1766.8) | |
| No qualifications | 82687 (14.2) | *5392* | *6.5%* | 48.5 (37.0-63.5) | |  | 469.9 (434.3-508.4) | |  | 3406.1 (3310.3-3504.7) | |
| **Tenure** |  |  |  |  |  |  |  |  |  |  |  |
| Owns in some capacity/ rent free | 295363 (50.8) | *5717* | *1.9%* | 27.7 (24.0-32.0) | |  | 216.1 (203.6-229.3) | |  | 2097.9 (2037.1-2160.6) | |
| Rents | 285846 (49.2) | *4649* | *1.6%* | 24.2 (21.4-27.5) | |  | 320.1 (301.2-340.2) | |  | 3114.8 (3011.4-3221.7) | |
| **Accommodation** |  |  |  |  |  |  |  |  |  |  |  |
| House | 288334 (49.6) | *5998* | *2.1%* | 29.8 (26.0-34.3) | |  | 234.9 (221.7-248.9) | |  | 2235.8 (2172.5-2300.9) | |
| Bedsit/ hotel/ caravan/ house share/ flat | 292875 (50.4) | *4368* | *1.5%* | 22.8 (20.0-26.0) | |  | 287.5 (270.1-306.1) | |  | 2827.2 (2730.3-2927.6) | |
| **Car ownership** |  |  |  |  |  |  |  |  |  |  |  |
| 1+ cars owned | 359732 (61.9) | *5254* | *1.5%* | 24.3 (21.3-27.7) | |  | 219.4 (207.7-231.8) | |  | 1877.6 (1818.4-1938.6) | |
| No cars owned | 221477 (38.1) | *5112* | *2.3%* | 27.4 (23.8-31.4) | |  | 344.9 (322.4-368.9) | |  | 3379.8 (3277.5-3485.4) | |

| **Supplementary Table 3:** Crude death rate per 100,000 person years stratified by age band among individuals with an SMI | | | | | | | | | | | | |  | |
| --- | --- | --- | --- | --- | --- | --- | --- | --- | --- | --- | --- | --- | --- | --- |
|  |  |  |  |  |  |  |  |  | **Age band** | |  |  |  |  |
|  |  |  |  |  |  | **15-44** |  |  | **45-64** |  |  | **65+** |  |  |
|  |  |  |  | ***Deaths (n, %)*** | | *51* |  |  | *179* |  |  | *205* |  |  |
|  |  | **N (%)** |  |  |  | **Rate (95% CI)** | |  | **Rate (95% CI)** | |  | **Rate (95% CI)** |  | |
| **Marital/ relationship status** |  |  |  |  |  |  |  |  |  |  |  |  |  |  |
| Currently or previously married | | 3313 (40.9) | | *248* | 7.5% | 146.2 (60.9-351.2) | |  | 1081.5 (871.0-1342.8) | | | 4803.7 (4116.2-5606.1) | | |
| Never married |  | 4785 (59.1) | | *187* | 3.9% | 369.9 (277.1-493.9) | |  | 1139.6 (933.9-1390.5) | | | 3555.6 (2646.0-4777.9) | | |
| **Social isolation** |  |  |  |  |  |  |  |  |  |  |  |  |  |  |
| Does not live alone |  | 5401 (66.7) | | *246* | 4.6% | 287.7 (205.6-402.7) | |  | 1009.5 (832.2-1224.5) | | | 4140.6 (3431.9-4995.7) | | |
| Live alone |  | 2697 (33.3) | | *189* | 7.0% | 421.0 (261.7-677.2) | |  | 1290.0 (1030.3-1615.3) | | | 4906.5 (4016.9-5993.0) | | |
| **Employment status** |  |  |  |  |  |  |  |  |  |  |  |  |  |  |
| Employed |  | 2211 (27.3) | | *48* | 2.2% | 221.9 (126.0-390.7) | |  | 673.3 (467.9-968.9) | |  | 1781.1 (849.1-3736.1) | | |
| Economically inactive |  | 5887 (72.7) | | *387* | 6.6% | 373.3 (272.8-510.9) | |  | 1272.5 (1084.4-1493.4) | | | 4718.7 (4105.2-5424.0) | | |
| **Qualifications** |  |  |  |  |  |  |  |  |  |  |  |  |  |  |
| Any education |  | 5983 (73.9) | | *219* | 3.7% | 290.2 (211.1-398.8) | |  | 904.0 (747.3-1093.6) | |  | 3097.3 (2470.0-3883.9) | | |
| No qualifications |  | 2115 (26.1) | | *216* | 10.2% | 471.2 (273.6-811.5) | |  | 1670.7 (1328.2-2101.5) | | | 5997.5 (5050.2-7122.4) | | |
| **Tenure** |  |  |  |  |  |  |  |  |  |  |  |  |  |  |
| Owns in some capacity/ rent free | | 2612 (32.3) | | *137* | 5.2% | 293.2 (176.7-486.3) | |  | 793.9 (580.0-1086.5) | |  | 4346.9 (3505.5-5390.3) | | |
| Rents |  | 5486 (67.7) | | *298* | 5.4% | 335.3 (241.8-464.8) | |  | 1252.0 (1060.9-1477.6) | | | 4552.8 (3812.6-5436.8) | | |
| **Accommodation** |  |  |  |  |  |  |  |  |  |  |  |  |  |  |
| House |  | 3075 (38.0) | | *172* | 5.6% | 282.2 (177.8-448.0) | |  | 1112.5 (870.8-1421.4) | | | 4527.4 (3682.4-5566.4) | | |
| Bedsit/ hotel/ caravan/ house share/ flat | | 5023 (62.0) | | *263* | 5.2% | 348.2 (247.5-489.8) | |  | 1112.0 (926.2-1335.0) | | | 4421.1 (3682.6-5307.7) | | |
| **Car ownership** |  |  |  |  |  |  |  |  |  |  |  |  |  |  |
| 1+ cars owned |  | 2856 (35.3) | | *120* | 4.2% | 282.7 (178.1-448.7) | |  | 843.5 (629.8-1129.7) | |  | 4079.8 (3147.0-5289.1) | | |
| No cars owned |  | 5242 (64.7) | | *315* | 6.0% | 347.8 (247.3-489.3) | |  | 1245.4 (1051.4-1475.2) | | | 4636.7 (3946.8-5447.2) | | |

## **Supplementary Figure 2:** Association of social exclusion (social participation indicators) with all-cause mortality; age, sex and ethnicity adjusted estimates, unweighted

***
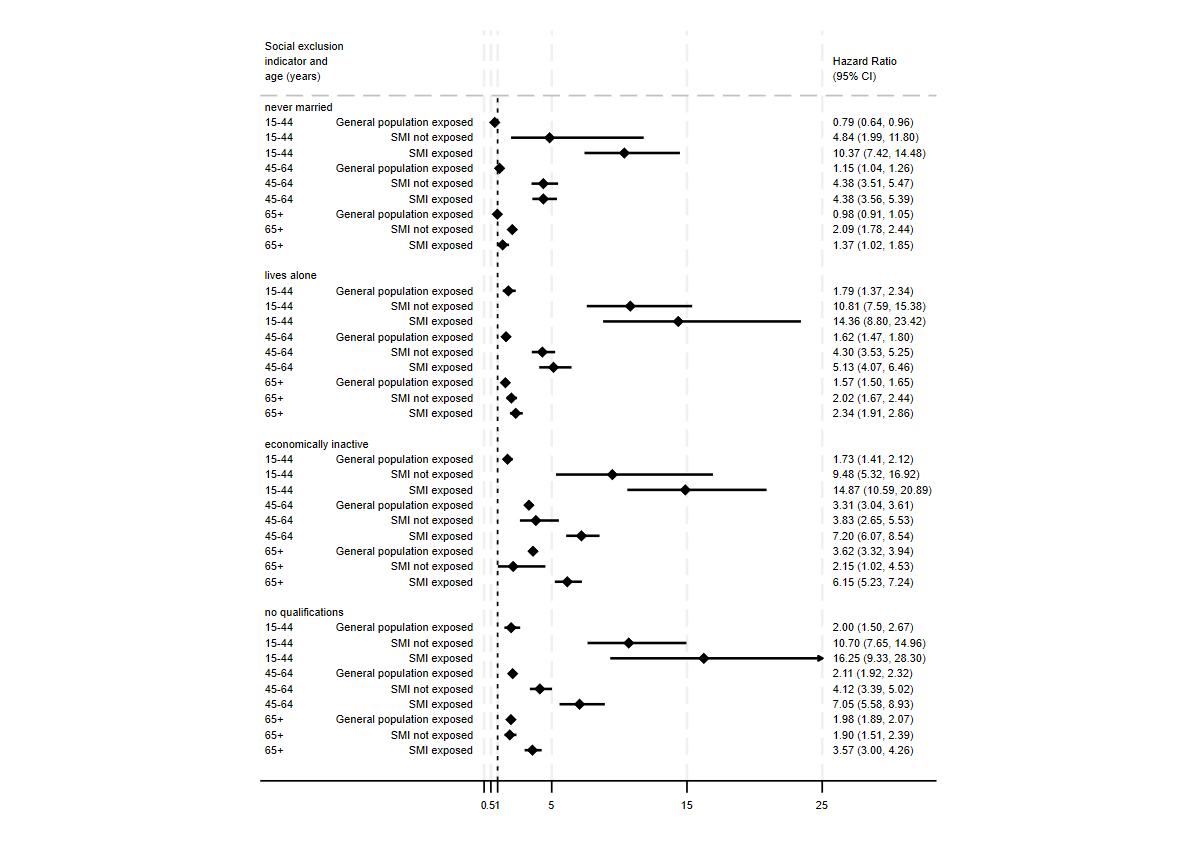
***

***Key:*** *Reference group across all models is the general population not exposed to any of the displayed social indicators, shown as the dashed reference line of 1.00. Displayed estimates are unweighted, stratified by age and adjusted for sex and ethnicity; *Social participation indicators were: marital/ relationship status, social isolation (living alone), economic inactivity, and lack of education participation (no qualifications).*

## **Supplementary Figure 3:** Association of social exclusion (material wealth indicators) with all-cause mortality; age, sex and ethnicity adjusted estimates, unweighted

**
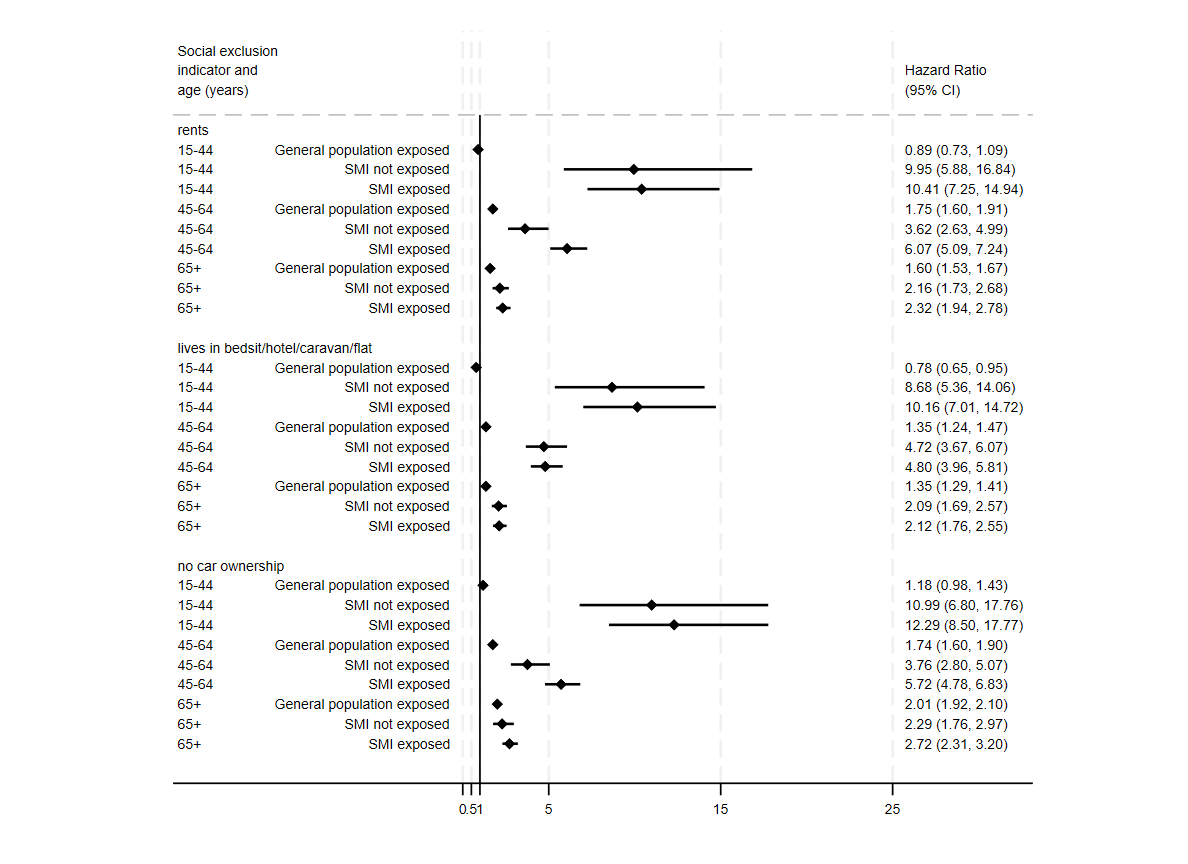
**

***Key:*** *Reference group across all models is the general population not exposed to any of the displayed social indicators, shown as the dashed reference line of 1.00. Displayed estimates are unweighted, stratified by age and adjusted for sex and ethnicity***.** *Material wealth indicators were: home ownership (renting), residential stability (lives in bedsit, hotel, caravan, house share), material assets (car ownership).*

## **Supplementary Figure 4**: Association of social exclusion (social participation indicators) with all-cause mortality; age, sex and ethnicity adjusted estimates, multiple imputation

**
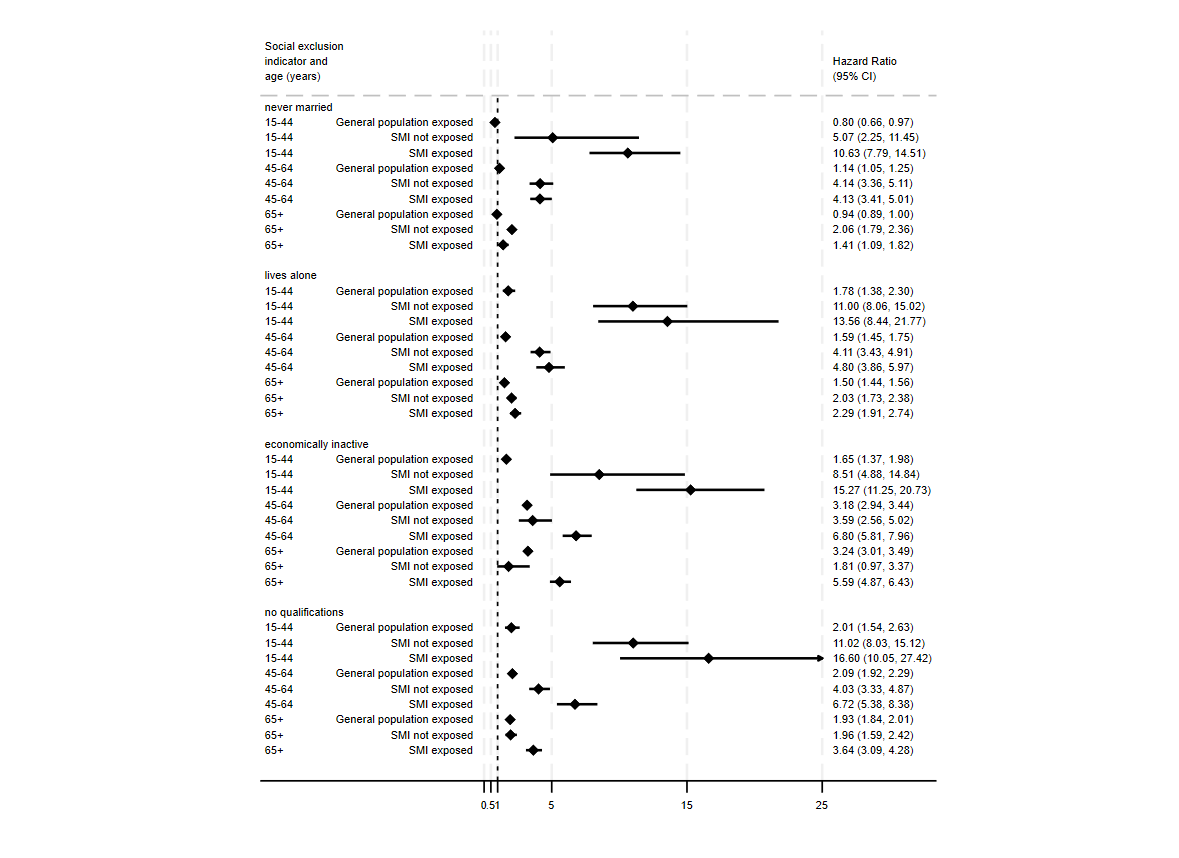
**

***Key:*** *Reference group across all models is the general population not exposed to any of the displayed social indicators, shown as the dashed reference line of 1.00. Displayed estimates were derived through multiple imputation, all estimates have been stratified by age and adjusted for sex and ethnicity; Social participation indicators were: marital/ relationship status, social isolation (living alone), economic inactivity, and lack of education participation (no qualifications).*

## **Supplementary Figure 5:** Association of social exclusion (material wealth indicators) with all-cause mortality; age, sex and ethnicity adjusted estimates, multiple imputation

**
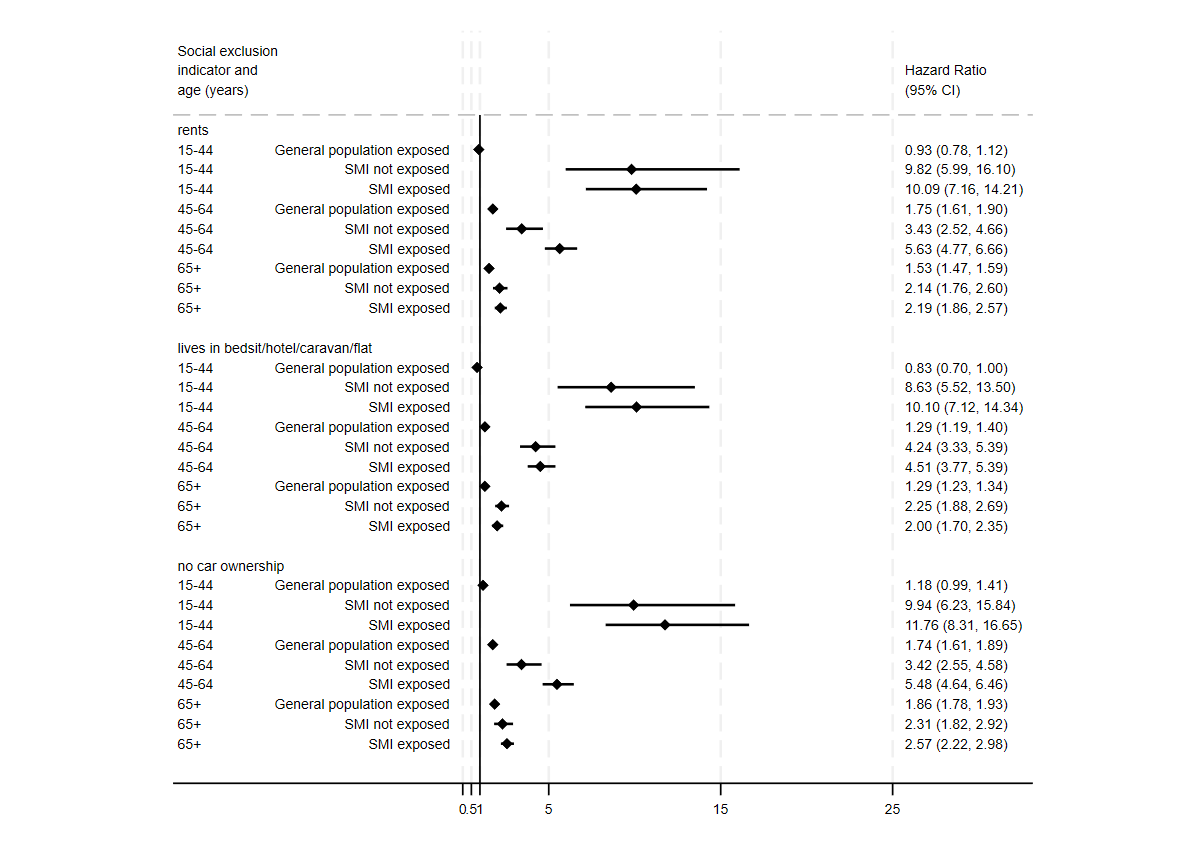
**

***Key:*** *Reference group across all models is the general population not exposed to any of the displayed social indicators, shown as the dashed reference line of 1.00. Displayed estimates were derived through multiple imputation, all estimates have been, stratified by age and adjusted for sex and ethnicity; Material wealth indicators were: home ownership (renting), residential stability (lives in bedsit, hotel, caravan, house share), material assets (car ownership).*

## **Supplementary Table 4:** Relative excess risk due to interaction (RERI) for social exclusion indicators with severe mental illness, RERI estimates with 95% Confidence Intervals (95% CI)

| **Age (years) and social exclusion indicator** | **Relative excess risk due to interaction (RERI)** | **95% CI** | **p value** |
| --- | --- | --- | --- |
| **15-44 years** |  |  |  |
| Never married | 8.06 | (2.63,13.49) | p<0.05 |
| Lives alone | 2.75 | (-5.01,10.52) | 0.49 |
| Economically inactive | 3.96 | (-2.44,10.36) | 0.22 |
| No qualifications | 3.95 | (-4.24,12.14) | 0.34 |
| Rents | -0.18 | (-6.08,5.72) | 0.95 |
| Lives in bedsit/hotel/caravan | 0.46 | (-4.31,5.23) | 0.85 |
| No car ownership | -0.17 | (-6.23,5.88) | 0.95 |
| **45-64 years** |  |  |  |
| Never married | 0.58 | (-0.80,1.96) | 0.41 |
| Lives alone | 0.20 | (-1.23,1.64) | 0.78 |
| Economically inactive | 1.05 | (-0.75,2.85) | 0.25 |
| No qualifications | 1.55 | (-0.13,3.23) | 0.07 |
| Rents | 1.97 | (0.44,3.50) | 0.01 |
| Lives in bedsit/hotel/caravan | -0.12 | (-1.59,1.36) | 0.88 |
| No car ownership | 1.23 | (-0.25,2.71) | 0.10 |
| **65+ years** |  |  |  |
| Never married | -0.45 | (-1.13,0.23) | 0.19 |
| Lives alone | -0.25 | (-0.85,0.36) | 0.42 |
| Economically inactive | 1.46 | (0.04,2.89) | 0.04 |
| No qualifications | 0.79 | (0.05,1.54) | 0.04 |
| Rents | -0.13 | (-0.84,0.58) | 0.72 |
| Lives in bedsit/hotel/caravan | -0.05 | (-0.72,0.61) | 0.87 |
| No car ownership | -0.35 | (-1.15,0.44) | 0.39 |

*Displayed estimates are adjusted for sex and ethnicity, and stratified by age*

## **Supplementary Table 5:** Association of severe mental illness (SMI) and social exclusion indicators with mortality, unadjusted (model 1) and age, sex adjusted associations (model 2)

|  | **Model 1:**  **Unadjusted/ crude** | | **Model 2: Adjusted ethnicity and sex, stratified by age** | | | |  |
| --- | --- | --- | --- | --- | --- | --- | --- |
|  | **HR (95% CI)** | | **HR (95% CI)** | |  | |  |
| **Age: 15-44 years** |  |  |  |  | |  | |
| SMI | 13.20 (9.82,17.75) | | 11.38 (8.45,15.33) | | | |  |
| Lives alone | 2.40 (1.85,3.12) | | 2.19 (1.69,2.85) | |  | |  |
| Never Married | 1.11 (0.91,1.36) | | 1.01 (0.82,1.23) | |  | |  |
| Economically inactive | 2.31 (1.90,2.81) | | 2.38 (1.93,2.94) | |  | |  |
| No qualifications | 2.52 (1.89,3.35) | | 2.47 (1.86,3.29) | |  | |  |
| Rents | 1.02 (0.84,1.24) | | 1.04 (0.85,1.27) | |  | |  |
| Lives in bedsit/hotel/caravan | 0.85 (0.70,1.03) | | 0.87 (0.72,1.06) | |  | |  |
| No car | 1.33 (1.09,1.61) | | 1.39 (1.14,1.69) | |  | |  |
|  |  |  |  |  | |  | |
| **Age: 45-64 years** |  |  |  |  | |  | |
| SMI | 4.25 (3.64,4.96) | | 4.09 (3.50,4.78) | |  | |  |
| Lives alone | 1.92 (1.74,2.12) | | 1.77 (1.60,1.95) | |  | |  |
| Never Married | 1.36 (1.24,1.49) | | 1.28 (1.16,1.40) | |  | |  |
| Economically inactive | 3.36 (3.09,3.66) | | 3.62 (3.33,3.94) | |  | |  |
| No qualifications | 2.26 (2.06,2.48) | | 2.21 (2.01,2.42) | |  | |  |
| Rents | 1.68 (1.55,1.83) | | 1.96 (1.80,2.15) | |  | |  |
| Lives in bedsit/hotel/caravan | 1.33 (1.22,1.44) | | 1.45 (1.33,1.58) | |  | |  |
| No car | 1.81 (1.66,1.97) | | 1.98 (1.82,2.16) | |  | |  |
|  |  |  |  |  | |  | |
| **Age: 65+ years** |  |  |  |  | |  | |
| SMI | 1.90 (1.66,2.19) | | 1.95 (1.70,2.24) | |  | |  |
| Lives alone | 1.60 (1.52,1.67) | | 1.58 (1.50,1.65) | |  | |  |
| Never Married | 1.04 (0.98,1.12) | | 0.98 (0.92,1.05) | |  | |  |
| Economically inactive | 3.43 (3.15,3.74) | | 3.64 (3.34,3.96) | |  | |  |
| No qualifications | 2.00 (1.91,2.09) | | 1.98 (1.90,2.08) | |  | |  |
| Rents | 1.49 (1.43,1.56) | | 1.60 (1.53,1.67) | |  | |  |
| Lives in bedsit/hotel/caravan | 1.27 (1.22,1.33) | | 1.35 (1.29,1.42) | |  | |  |
| No car | 1.80 (1.73,1.89) | | 2.01 (1.92,2.10) | |  | |  |

***Key:*** *Displayed estimates are weighted for non-linkage, and display associations with no interactions. HR (95% CI): Hazard Ratio with 95% Confidence Intervals*

## **Supplementary Table 6:** Sensitivity analyses, E-values*

| **Age (years)** | **Social exclusion indicator** |  | **HR** | **(95% CI)** | | **E-Value HR** | **E value**  **(Lower 95% CI)** |
| --- | --- | --- | --- | --- | --- | --- | --- |
| 15-44 | never married | General population, not exposed | 1.00 | REF |  | n/a | n/a |
| 15-44 | never married | General population exposed | 0.79 | (0.65 | 0.97) | 1.84 | 1.21 |
| 15-44 | never married | SMI not exposed | 4.79 | (1.96 | 11.67) | 9.05 | 3.34 |
| 15-44 | never married | SMI exposed | 10.94 | (7.76 | 15.43) | 21.37 | 15.00 |
| 45-64 | never married | General population, not exposed | 1.00 | REF |  | n/a | n/a |
| 45-64 | never married | General population exposed | 1.15 | (1.05 | 1.26) | 1.57 | 1.27 |
| 45-64 | never married | SMI not exposed | 4.36 | (3.47 | 5.46) | 8.18 | 6.40 |
| 45-64 | never married | SMI exposed | 4.20 | (3.40 | 5.17) | 7.86 | 6.26 |
| 65+ | never married | General population, not exposed | 1.00 | REF |  | n/a | n/a |
| 65+ | never married | General population exposed | 0.98 | (0.91 | 1.05) | 1.17 | 1.00 |
| 65+ | never married | SMI not exposed | 2.16 | (1.85 | 2.53) | 3.75 | 3.10 |
| 65+ | never married | SMI exposed | 1.43 | (1.06 | 1.92) | 2.21 | 1.33 |
| 15-44 | lives alone | General population, not exposed | 1.00 | REF |  | n/a | n/a |
| 15-44 | lives alone | General population exposed | 1.79 | (1.37 | 2.34) | 2.98 | 2.09 |
| 15-44 | lives alone | SMI not exposed | 11.57 | (8.07 | 16.58) | 22.62 | 15.62 |
| 15-44 | lives alone | SMI exposed | 13.94 | (8.46 | 22.97) | 27.37 | 16.40 |
| 45-64 | lives alone | General population, not exposed | 1.00 | REF |  | n/a | n/a |
| 45-64 | lives alone | General population exposed | 1.63 | (1.47 | 1.80) | 2.64 | 2.30 |
| 45-64 | lives alone | SMI not exposed | 4.24 | (3.46 | 5.20) | 7.95 | 6.38 |
| 45-64 | lives alone | SMI exposed | 4.90 | (3.88 | 6.20) | 9.28 | 7.21 |
| 65+ | lives alone | General population, not exposed | 1.00 | REF |  | n/a | n/a |
| 65+ | lives alone | General population exposed | 1.57 | (1.50 | 1.65) | 2.52 | 2.36 |
| 65+ | lives alone | SMI not exposed | 2.07 | (1.71 | 2.51) | 3.57 | 2.82 |
| 65+ | lives alone | SMI exposed | 2.43 | (1.99 | 2.96) | 4.29 | 3.39 |
| 15-44 | economically inactive | General population, not exposed | 1.00 | REF |  | n/a | n/a |
| 15-44 | economically inactive | General population exposed | 1.74 | (1.41 | 2.14) | 2.87 | 2.18 |
| 15-44 | economically inactive | SMI not exposed | 9.77 | (5.37 | 17.77) | 19.02 | 10.21 |
| 15-44 | economically inactive | SMI exposed | 15.49 | (10.99 | 21.84) | 30.47 | 21.46 |
| 45-64 | economically inactive | General population, not exposed | 1.00 | REF |  | n/a | n/a |
| 45-64 | economically inactive | General population exposed | 3.29 | (3.02 | 3.59) | 6.04 | 5.49 |
| 45-64 | economically inactive | SMI not exposed | 3.69 | (2.54 | 5.37) | 6.84 | 4.51 |
| 45-64 | economically inactive | SMI exposed | 6.95 | (5.84 | 8.27) | 13.39 | 11.16 |
| 65+ | economically inactive | General population, not exposed | 1.00 | REF |  | n/a | n/a |
| 65+ | economically inactive | General population exposed | 3.61 | (3.31 | 3.94) | 6.68 | 6.08 |
| 65+ | economically inactive | SMI not exposed | 2.30 | (1.09 | 4.86) | 4.03 | 1.40 |
| 65+ | economically inactive | SMI exposed | 6.33 | (5.38 | 7.45) | 12.14 | 10.23 |
| 15-44 | no qualifications | General population, not exposed | 1.00 | REF |  | n/a | n/a |
| 15-44 | no qualifications | General population exposed | 2.01 | (1.50 | 2.68) | 3.43 | 2.37 |
| 15-44 | no qualifications | SMI not exposed | 10.94 | (7.75 | 15.44) | 21.37 | 14.99 |
| 15-44 | no qualifications | SMI exposed | 17.57 | (10.08 | 30.63) | 34.63 | 19.64 |
| 45-64 | no qualifications | General population, not exposed | 1.00 | REF |  | n/a | n/a |
| 45-64 | no qualifications | General population exposed | 2.11 | (1.92 | 2.32) | 3.65 | 3.26 |
| 45-64 | no qualifications | SMI not exposed | 4.03 | (3.30 | 4.93) | 7.53 | 6.05 |
| 45-64 | no qualifications | SMI exposed | 6.77 | (5.33 | 8.61) | 13.03 | 10.13 |
| 65+ | no qualifications | General population, not exposed | 1.00 | REF |  | n/a | n/a |
| 65+ | no qualifications | General population exposed | 1.98 | (1.89 | 2.07) | 3.38 | 3.19 |
| 65+ | no qualifications | SMI not exposed | 1.96 | (1.56 | 2.46) | 3.33 | 2.49 |
| 65+ | no qualifications | SMI exposed | 3.66 | (3.07 | 4.37) | 6.78 | 5.59 |
| 15-44 | rents | General population, not exposed | 1.00 | REF |  | n/a | n/a |
| 15-44 | rents | General population exposed | 0.89 | (0.73 | 1.09) | 1.50 | 1.00 |
| 15-44 | rents | SMI not exposed | 9.53 | (5.53 | 16.40) | 18.55 | 10.53 |
| 15-44 | rents | SMI exposed | 11.15 | (7.72 | 16.09) | 21.79 | 14.92 |
| 45-64 | rents | General population, not exposed | 1.00 | REF |  | n/a | n/a |
| 45-64 | rents | General population exposed | 1.74 | (1.59 | 1.90) | 2.88 | 2.56 |
| 45-64 | rents | SMI not exposed | 3.60 | (2.60 | 4.98) | 6.66 | 4.64 |
| 45-64 | rents | SMI exposed | 5.79 | (4.84 | 6.92) | 11.06 | 9.15 |
| 65+ | rents | General population, not exposed | 1.00 | REF |  | n/a | n/a |
| 65+ | rents | General population exposed | 1.60 | (1.52 | 1.67) | 2.58 | 2.41 |
| 65+ | rents | SMI not exposed | 2.23 | (1.79 | 2.78) | 3.89 | 2.98 |
| 65+ | rents | SMI exposed | 2.40 | (2.01 | 2.87) | 4.23 | 3.43 |
| 15-44 | lives in bedsit/hotel/caravan | General population, not exposed | 1.00 | REF |  | n/a | n/a |
| 15-44 | lives in bedsit/hotel/caravan | General population exposed | 0.78 | (0.65 | 0.95) | 1.88 | 1.29 |
| 15-44 | lives in bedsit/hotel/caravan | SMI not exposed | 9.28 | (5.68 | 15.15) | 18.05 | 10.84 |
| 15-44 | lives in bedsit/hotel/caravan | SMI exposed | 10.34 | (7.09 | 15.09) | 20.17 | 13.66 |
| 45-64 | lives in bedsit/hotel/caravan | General population, not exposed | 1.00 | REF |  | n/a | n/a |
| 45-64 | lives in bedsit/hotel/caravan | General population exposed | 1.34 | (1.23 | 1.47) | 2.02 | 1.76 |
| 45-64 | lives in bedsit/hotel/caravan | SMI not exposed | 4.63 | (3.58 | 5.99) | 8.73 | 6.62 |
| 45-64 | lives in bedsit/hotel/caravan | SMI exposed | 4.63 | (3.82 | 5.63) | 8.73 | 7.10 |
| 65+ | lives in bedsit/hotel/caravan | General population, not exposed | 1.00 | REF |  | n/a | n/a |
| 65+ | lives in bedsit/hotel/caravan | General population exposed | 1.34 | (1.28 | 1.41) | 2.02 | 1.88 |
| 65+ | lives in bedsit/hotel/caravan | SMI not exposed | 2.16 | (1.75 | 2.66) | 3.74 | 2.90 |
| 65+ | lives in bedsit/hotel/caravan | SMI exposed | 2.19 | (1.82 | 2.64) | 3.80 | 3.04 |
| 15-44 | no car ownership | General population, not exposed | 1.00 | REF |  | n/a | n/a |
| 15-44 | no car ownership | General population exposed | 1.18 | (0.97 | 1.43) | 1.64 | 1.00 |
| 15-44 | no car ownership | SMI not exposed | 11.11 | (6.81 | 18.13) | 21.71 | 13.10 |
| 15-44 | no car ownership | SMI exposed | 12.91 | (8.87 | 18.77) | 25.31 | 17.23 |
| 45-64 | no car ownership | General population, not exposed | 1.00 | REF |  | n/a | n/a |
| 45-64 | no car ownership | General population exposed | 1.74 | (1.59 | 1.90) | 2.88 | 2.56 |
| 45-64 | no car ownership | SMI not exposed | 3.75 | (2.78 | 5.07) | 6.96 | 5.00 |
| 45-64 | no car ownership | SMI exposed | 5.47 | (4.56 | 6.55) | 10.41 | 8.59 |
| 65+ | no car ownership | General population, not exposed | 1.00 | REF |  | n/a | n/a |
| 65+ | no car ownership | General population exposed | 2.00 | (1.91 | 2.10) | 3.41 | 3.23 |
| 65+ | no car ownership | SMI not exposed | 2.34 | (1.80 | 3.05) | 4.11 | 3.00 |
| 65+ | no car ownership | SMI exposed | 2.80 | (2.38 | 3.31) | 5.05 | 4.19 |

***Key: *****Displayed E-values HR and lower e-value 95% confidence intervals display the minimum effect size for an unmeasured confounder (between displayed exposures and all-cause mortality outcomes) to ‘fully explain’ away observed associations*

## **Supplementary Table 7:** Sensitivity analyses, association between economic inactivity and mortality

|  |  | **Economic inactivity, (including students and retirees)** | | |  | **Economic inactivity,**  **(excluding students and retirees)** | | |
| --- | --- | --- | --- | --- | --- | --- | --- | --- |
| **Age** |  | **HR** | **(95% CI)** | |  | **HR** | **(95% CI)** | |
| 16-44 | General population/ not economically inactive | 1.00 | REF |  |  | 1.00 | REF |  |
| 16-44 | General population, economically inactive | 1.74 | (1.41 | 2.14) |  | 2.48 | (1.99 | 3.10) |
| 16-44 | SMI/ not economically inactive | 9.77 | (5.37 | 17.77) |  | 9.66 | (5.31 | 17.57) |
| 16-44 | SMI, economically inactive | 15.49 | (10.99 | 21.84) |  | 16.68 | (11.74 | 23.72) |
| 45-64 | General population/ not economically inactive | 1.00 | REF |  |  | 1.00 | REF |  |
| 45-64 | General population, economically inactive | 3.29 | (3.02 | 3.59) |  | 3.17 | (2.88 | 3.49) |
| 45-64 | SMI/ not economically inactive | 3.69 | (2.54 | 5.37) |  | 3.69 | (2.54 | 5.37) |
| 45-64 | SMI, economically inactive | 6.95 | (5.84 | 8.27) |  | 6.96 | (5.80 | 8.34) |
| 65+ | General population/ not economically inactive | 1.00 | REF |  |  | 1.00 | REF |  |
| 65+ | General population, economically inactive | 3.61 | (3.31 | 3.94) |  | 3.23 | (2.83 | 3.69) |
| 65+ | SMI/ not economically inactive | 2.30 | (1.09 | 4.86) |  | 2.25 | (1.07 | 4.74) |
| 65+ | SMI, economically inactive | 6.33 | (5.38 | 7.45) |  | 5.18 | (3.56 | 7.54) |

| **Supplementary Table 8: Developing a social exclusion indicator from census measures** Many previous commentators have highlighted the complexity of measuring the concept of social exclusion^[1]^. Social exclusion has been defined as occurring when individuals are prevented from fully participating in society by external forces, not by choice^[1, 2]^. Conceptually, social exclusion spans multiple dimensions which may include impaired participation in work, education, leisure and citizenship activities, alongside impaired social relationships and social isolation, as well as housing deprivation, poverty and material wealth^[3]^. Within the context of mental health, whereas ‘inclusion’ may denote the involvement of people with severe mental illnesses in mainstream society, ‘social exclusion’ leads to people being excluded from mainstream society through underlying processes, operating through stigma and discrimination^[1]^.  Therefore, the concept of social exclusion overlaps with, and may comprise, material indicators of disadvantage^[1-3]^. Although material disadvantage and poverty may play a role in increasing risks for social exclusion, poverty and material disadvantage indicators on their own are not sufficient as a measure of social exclusion^[2]^. Most conceptualisations of social exclusion also include measures relating to social relationships as well as indicators of poverty and material wealth^[1]^.  Based on previous work in this area, we aimed to develop a dimensional indicator using measures from the Census from England, which would map to underlying domains that had previously been identified as relevant to the social exclusion construct, in the wider literature^[1]^. Specifically, we attempted to map available indicators from census to proposed domains for social exclusion which had been developed and presented in a previous review^[1]^ and in a study using empirical data from the UK, which had also been developed a formulation of social exclusion domains with the input of an expert advisory panel that had comprised practitioners, academic experts and lay members^[3]^.  **Table 1: Mapping of census indicators to social exclusion domains identified in the literature**   \| **Social exclusion domains identified from the literature, relevant to mental health** \| \| \|  \| \| --- \| --- \| --- \| --- \| \| **From Dykhoorn et al, 2024^[3]^** \| ***From Morgan et al, 2007^[1]^**  **Centre for the Analysis of Social Exclusion, LSE** \| ****From Morgan et al, 2007^[1]^**  **Social exclusion indicator from PSE Survey** \| **Mapped to relevant census indicator used in the present study** \| \| **Material exclusion,** “inc. economic strain, inadequate housing, lack of possession of material assets” \| **Consumption:** “The capacity to purchase goods and services” \| “Impoverishment or exclusion from adequate sources of income” \| Car ownership, housing ownership (own/ rents), housing stability \| \| Employment and education included under the ‘Material exclusion’ domain \| **Production: “**Participation in economically or socially valuable activities” \| “Labour market exclusion, Service exclusion” \| Economic inactivity, Education participation (highest qualification) \| \| **Political exclusion, “**unable to participate in political processes, collective action, local/ regional/ national decision-making” \| **Political engagement: “**Involvement in local or national decision making” \| None proposed \| None available in census \| \| **Digital exclusion, “**barriers to accessing digital information due to lack of access to technology or low IT literacy” \| None proposed \| None proposed \| None available in census \| \| **Relational exclusion,** “lack of meaningful social and close relationships, loneliness and social isolation” \| **Social interaction:** “Integration with family, friends, and community” \| “Exclusion from social relations” \| Marital/ relationship status; Social isolation (lives alone) \| \| **Structural exclusion, “**exclusion due to lack of access to power and through discriminatory processes and stigma” \| None proposed \| None proposed \| Indicators for social status which may map to structural disadvantage (eg. gender, ethnicity) available in census as indirect measure. SMI status, measured through health records, also available- people with SMI are known to experience high levels of stigma and structural discrimination^[4]^ \|   ***Key:*** *Adapted from Morgan et al, 2007^[1]^; *from the Centre for the Analysis of Social Exclusion, LSE, London^[5]^; **from the Poverty Social Exclusion (PSE) survey, Pantazis et al 1999^[6]^.*  Prior literature has also highlighted that social exclusion measures need to incorporate a notion of relative poverty and material disadvantage leading to people being excluded from mainstream society, and this plays a role in the processes of exclusion^[1]^. Thus, the *“relational processes by which groups are excluded*” is an agreed underlying key feature of social exclusion^[2]^.  The principal components analysis (PCA) approach taken in the study enabled data reduction into a single dimensional scale, which gave a larger weight to those measures most unequally distributed in the study population as a whole^[7]^.  Please see the statistical methods section of the manuscript for more detail.  **References**  1. Morgan, C., et al., *Social exclusion and mental health: conceptual and methodological review.* Br J Psychiatry, 2007. **191**: p. 477-83.  2. Cuesta, J., B. López-Noval, and M. Niño-Zarazúa, *Social exclusion concepts, measurement, and a global estimate.* PLOS ONE, 2024. **19**(2): p. e0298085.  3. Dykxhoorn, J., et al., *Measuring social exclusion and its distribution in England.* Social Psychiatry and Psychiatric Epidemiology, 2024. **59**(1): p. 187-198.  4. Thornicroft, G., et al., *Global pattern of experienced and anticipated discrimination against people with schizophrenia: a cross-sectional survey.* Lancet, 2009. **373**(9661): p. 408-15.  5. Burchardt T, *Social exclusion*, in *The Blackwell encyclopaedia of social work*, M. Davies and R. Barton, Editors. 2000, Blackwell: Malden, Mass. ;. p. 320-321.  6. Pantazis, C., D. Gordon, and R. Levitas, *Poverty and social exclusion in Britain: The millennium survey*. 2006, Great Britain: The policy press.  7. Vyas, S. and L. Kumaranayake, *Constructing socio-economic status indices: how to use principal components analysis.* Health Policy and Planning, 2006. **21**(6): p. 459-468. |
| --- | --- | --- | --- | --- | --- | --- | --- | --- | --- | --- | --- | --- | --- | --- | --- | --- | --- | --- | --- | --- | --- | --- | --- | --- | --- | --- | --- | --- | --- | --- | --- | --- |
